# Kinetic Evaluation of the Production of Mead from a Non-*Saccharomyces* Strain

**DOI:** 10.3390/foods13121948

**Published:** 2024-06-20

**Authors:** Jorge Alberto Jose-Salazar, Christian Bryan Ballinas-Cesatti, Diana Maylet Hernández-Martínez, Eliseo Cristiani-Urbina, Guiomar Melgar-Lalanne, Liliana Morales-Barrera

**Affiliations:** 1 Departamento de Ingeniería Bioquímica, Escuela Nacional de Ciencias Biológicas, Instituto Politécnico Nacional, Av. Wilfrido Massieu s/n, Unidad Profesional Adolfo López Mateos, Ciudad de México 07738, Mexico; jorgeajosesalazar@gmail.com (J.A.J.-S.); christian.bryan.ballinas.cesatti@hotmail.com (C.B.B.-C.); ecristianiu@yahoo.com.mx (E.C.-U.); 2 Departamento de Biofísica, Escuela Nacional de Ciencias Biológicas, Instituto Politécnico Nacional, Prolongación de Carpio y Plan de Ayala s/n, Col. Santo Tomás, Ciudad de México 11340, Mexico; dhernandezmar@ipn.mx; 3 Centro de Investigaciones Biomédicas, Universidad Veracruzana, Av. Castelazo Anaya s/n, Industrial Ánimas, Xalapa 91190, Veracruz, Mexico; gmelgar@uv.mx

**Keywords:** honey mead, *Pichia kudriavzevii*, non-*Saccharomyces* fermentation, FTIR, sensorial analysis, antioxidant activity

## Abstract

There is a growing market for craft beverages with unique flavors. This study aimed to obtain a palate-pleasing mead derived from *Pichia kudriavzevii* 4A as a monoculture. Different culture media were evaluated to compare the fermentation kinetics and final products. The crucial factors in the medium were ~200 mg L^−1^ of yeast assimilable nitrogen and a pH of 3.5–5.0. A panel of judges favored the mead derived from *Pichia kudriavzevii* 4A (fermented in a medium with honey initially at 23 °Bx) over a commercial sample produced from *Saccharomyces cerevisiae*, considering its appearance, fruity and floral flavors (provided by esters, aldehydes, and higher alcohols), and balance between sweetness (given by the 82.91 g L^−1^ of residual sugars) and alcohol. The present mead had an 8.57% *v*/*v* ethanol concentration, was elaborated in 28 days, and reached a maximum biomass growth (2.40 g L^−1^) on the same fermentation day (6) that the minimum level of pH was reached. The biomass growth yield peaked at 24 and 48 h (~0.049 g g^−1^), while the ethanol yield peaked at 24 h (1.525 ± 0.332 g g^−1^), in both cases declining thereafter. The Gompertz model adequately describes the kinetics of sugar consumption and the generation of yeast biomass and ethanol. Pathogenic microorganisms, methanol, lead, and arsenic were absent in the mead. Thus, *Pichia kudriavzevii* 4A produced a safe and quality mead with probable consumer acceptance.

## 1. Introduction

Mead is traditionally obtained from the uncontrolled fermentation of an aqueous solution of honey, usually with *Saccharomyces* yeast strains. It was probably one of the first alcoholic drinks fermented by humans, given that honey has been gathered and eaten since ancient times. Cave paintings in Valencia, Spain depict the gathering of honey and are estimated to be 8000 to 15000 years old [1,2]. Honey is a viscous liquid mainly consisting of carbohydrates (80–85%), water (15–17%), and proteins (0.3%), with lesser amounts of pigments, phenols, vitamins, and minerals [3]. Its annual worldwide production in 2021 was estimated at 1.77 million metric tons [4].

Mead very commonly includes fruit (especially grapes and apples) and may also have herbs, flowers, and spices. The final ethanol content ranges from 8 to 18% *v*/*v* [5,6]. Beneficial antioxidant properties, such as anti-thrombotic, anti-ischemic, and vasorelaxant effects, have been attributed to mead [7].

*Saccharomyces*, *Schizosaccharomyces*, and *Zygosaccharomyces* are among the yeasts existing in honey and are often identified in the products of uncontrolled fermentation. Strains of the genera *Debaryomyces*, *Hansenula*, *Lipomyces*, *Pichia*, and *Torulaspora* have been detected as well [8]. In addition to generating ethanol from carbohydrates, these yeasts confer fruity and floral flavors and aromas to the beverage [9].

The fabrication of mead was in decline during many years because its artisanal process implied a lack of uniformity in the quantity and quality of the beverage, sometimes causing unpleasant flavors [10]. These problems have been attributed to diverse factors, including the origin of the honey, the temperature of fermentation, and the composition of the mead [11]. The control of these factors has led to a recovery of the market share of mead [6], evidenced by total sales of USD 487.9 million in 2021 [12]. However, a challenge remains in fermenting and maturing a satisfactory product in less than the 3–6 months currently required. Such an achievement would lead to a greater availability and lower price [5,6,10].

Many recent studies on mead production have focused on improving its sensory characteristics (e.g., by adding fruit) as well as on increasing the concentration of ethanol [13,14]. Other works have analyzed factors possibly inhibiting the fermentation process of mead, treatments for diminishing the initial microbial load of the wort, changes in antioxidant activity, and the formation of furfural compounds [15,16,17]. Some authors have attempted to improve the conditions of fermentation through the addition of salts and vitamins to the formulation of the culture medium to reduce the stress sensitivity of the yeast. Volatile acidity has also been taken into account, since it influences the quantity and quality of the beverage [18,19,20,21]. The proportion of yeast assimilable nitrogen (YAN) has been increased in the medium to try to compensate for nutritional deficiencies and thus optimize the metabolism of the yeast, leading to a greater yield of mead and improved sensory properties (color, aroma, and flavor) [22,23].

Another critical factor is the selection of the starter culture [24], which is critical for the attainment of distinct flavors and aromas of the final product. In this regard, the few studies on the preparation of mead with non-*Saccharomyces* yeasts have evaluated *Torulaspora delbrueckii*, *Kluyveromyces thermotolerans*, Pichia jadinii, and *K. lactis* [9,25]. Hence, further research is necessary on the quantitative and qualitative aspects of the production of mead with non-*Saccharomyces* yeasts that are innocuous, resistant to the harsh conditions of fermentation (e.g., the high osmotic pressure, high ethanol concentration, and low pH), and capable of generating a palate-pleasing beverage with an acceptable yield in a relatively short time.

Our group previously isolated a non-*Saccharomyces* yeast, the *Pichia kudriavzevii* 4A strain [26], and used it to obtain malt beer [27]. *P. kudriavzevii* has gained biotechnological importance due to its tolerance to different operating factors [28] that confer it various technological benefits, such as its tolerance to osmotic pressure (48% *w*/*v* in glucose), ethanol (up to 20% *v*/*v*), temperature (5–45 °C), and furfural compounds (7 g L^−1^ *w*/*v* 5-HMF) [26,28,29]. These conditions offer *P. kudriavzevii* a competitive advantage by reducing the possibility of cross-contamination as well as the cooling costs of fermenters (especially important in tropical countries) [30].

*P. kudriavzevii* has also been used to obtain rice beer (“gora”), apple cider, and wines from grapes, oranges, and apricots. Various reports have confirmed that this strain provides aromatic profiles that are not generated by *Saccharomyces* yeasts [27,28,31,32,33,34]. Other *Pichia* species, such as *P. jadinii* and *P. kluyveri* PkCid1, have been studied to obtain mead and cider, respectively [9,35]. However, to the best of our knowledge, there are no previous reports on the kinetic behavior of *P. kudriavzevii* in mead production and the importance of YAN in obtaining a good-quality mead with this strain.

Therefore, the current contribution aimed to evaluate the production of mead by using *P. kudriavzevii* 4A as the starter culture. After inoculating this strain into different formulations of honey-based culture media, the resulting biomass yield, substrate consumption, ethanol concentration, and phenolic compound content were assessed during the process of fermentation and maturation. Subsequently, the mead was subjected to physicochemical and microbiological analysis to determine whether it complied with the parameters of consumer safety and a sensory test to ascertain whether it could achieve consumer acceptance. The present study was able to obtain control over the fermentation process to ensure the production of a palate-pleasing mead that is safe for consumption. Moreover, it was produced in a relatively short period of fermentation.

## 2. Materials and Methods

### 2.1. Honey

The clear, monofloral honey used in this study was produced from orange trees (*Citrus sinensis*) in Teocelo, Veracruz, Mexico (19.392173, −96.979410). After being collected, it was stored at room temperature.

### 2.2. Yeast

*P. kudriavzevii* 4A was isolated from the pericarp of sugarcane collected in Úrsulo Galvan, Veracruz, Mexico and subjected to molecular identification, as reported by Nieto-Sarabia et al. [26]. It was conserved in YPG agar medium, with 20 g L^−1^ bacteriological agar, 20 g L^−1^ dextrose, 10 g L^−1^ yeast extract, and 20 g L^−1^ casein peptone (BD Bioxon, Cuautitlán Izcalli, Mexico State, Mexico).

### 2.3. The Honey-Based Culture Media

The control medium (M0) consisted of a solution of honey with 317 g L^−1^, implying 23 degrees Brix (°Bx). The first enriched medium (M1) was a solution of honey with a robust mineral content (3.0 g (NH_4_)_2_SO_4_ L^−1^, 1.0 g KH_2_PO_4_ L^−1^, 0.3 g MgSO_4_·7H_2_O L^−1^, 0.1 g KCl L^−1^, 0.05 g CaCl_2_ L^−1^, and 0.001 g FeCl_3_ L^−1^) (JT Baker, Xalostoc, Mexico State, Mexico; purity >99%) plus 1.0 g L^−1^ of yeast extract (BD Bioxon, Cuautitlán Izcalli, Mexico State, Mexico). The three different concentrations of honey tested (15, 19, and 23 °Bx) were based on 203, 256, and 317 g L^−1^ and were denominated M1–15, M1–19, and M1–23, respectively. The second enriched medium (M2) was a solution of honey with 1.0 g L^−1^ (NH_4_)_2_SO_4_, 0.5 g L^−1^ KH_2_PO_4_, and 1.0 g L^−1^ yeast extract. The two concentrations of honey tested (19 and 23 °Bx) were denominated M2–19 and M2–23, respectively. The honey-based media were subjected to a pasteurization process (heating to 90 °C for 30 min in a water bath followed by an ice bath for 15 min) with the aim of reducing the microbiological load [16]. They were then stored under refrigeration (at 6 °C) until use.

### 2.4. Preparation of the Inoculum

The inoculum was prepared by cultivating *P. kudriavzevii* 4A in YPG liquid medium incubated at room temperature (30 ± 2 °C) under constant agitation (120 rpm) for 48 h. Subsequently, 1 mL of this suspension was transferred to distinct Erlenmeyer flasks with either the M0, M1, or M2 medium and incubated under the previously mentioned conditions. After 48 h, the suspension was centrifuged at 1917× *g* for 20 min to obtain the cell pellet, to which sterile type II deionized water (0.7–1.0 mmhos cm^−1^) was added before centrifuging again. The same procedure was performed two more times to afford *P. kudriavzevii* 4A free of the culture medium. To the yeast, a known quantity of sterile type II deionized water was added, and the biomass was determined (in dry weight) to establish the cellular concentration of the suspension. With these data, the suspension volume needed in the distinct media (M0, M1–15, M1–19, M1–23, M2–19, and M2–23) was calculated.

### 2.5. Evaluation of Fermentation in Different Culture Media to Produce Mead

To 500 mL Erlenmeyer flasks, 300 mL of the control medium (M0) was added, followed by the inoculation of 0.4 g L^−1^ of *P. kudriavzevii* 4A. Then, previously sanitized airlocks with Star San (Five Star, Arvada, CO, USA) were installed on the flasks, which were incubated at 30 °C for 28 days. During fermentation, samples of 2 mL were taken at different experimental times. The biomass concentration was determined by measuring the dry cell weight. The culture samples were filtered under vacuum through pre-weighed Whatman GF/A membranes (1.6 μm, Cytiva, St. Louis, MO, USA), which were washed twice with sterile type II deionized water and subsequently dried to a constant weight at 60 °C. The biomass concentration was calculated using the weight differential of the membrane before and after filtration and drying. The filtrates were used to determine the pH, °Bx, and concentration of the residual substrate and ethanol.

The biomass growth, the consumption of the substrate, and the ethanol production were calculated by means of Equations (1)–(3).

(1) $$Biomass\;growth=X_{t}-X_{0}$$

(2) $$Consumption\;of\;the\;substrate=S_{0}-S_{t}$$

(3) $$Ethanol\;production=P_{t}-P_{0}$$

where *X*_0_, *S*_0_, and *P*_0_ represent the concentrations of the biomass, substrate (e.g., reducing sugars), and ethanol (g L^−1^), respectively, at time 0 (*t*_0_ = day 0), while *X_t_*, *S_t_*, and *P_t_* correspond to the concentrations of the biomass, residual substrate, and ethanol, respectively, at time *t* (in days).

In accordance with the results obtained with M0, the medium was reformulated to create an enriched medium (M1), which was prepared with honey at either 15, 19, or 23 °Bx and subjected to a kinetic study under the aforementioned conditions. The necessary analytical determinations were made for M1, leading to another reformulation of the enriched medium to yield M2, which was elaborated with either 19 or 23 °Bx of honey and subjected to a kinetic evaluation. The two beverages obtained with M2 were filtered with a Whatman GF/A membrane, and the filtrate was conserved in sterile Erlenmeyer flasks under refrigeration (at 6 °C) for the posterior sensory test.

Once the sensory test established whether M2–19 or M2–23 was favored by the panel of judges, the antioxidant capacity of the preferred beverage was examined during fermentation. This beverage was also subjected to physicochemical and microbiological analysis to identify its characteristics and compare them to those of the meads reported in the literature.

### 2.6. Sensory Testing

The sensory tests (sensory profile and overall preference) were carried out with the participation of 46 untrained judges between 18 and 50 years of age. They first compared the products derived from M2–19 and M2–23 (based on *P. kudriavzevii* 4A as the starter culture) and then compared the preferred beverage (derived from M2–23) with a commercially produced mead (made with *Saccharomyces cerevisiae*). All samples were evaluated at 6 °C. These two tests were conducted with the same recently elaborated mead. Additionally, the judges performed a profile test, examining the appearance (color and clarity), taste (sweet, acidic, and bitter), and aroma (fruity, woody, alcoholic, herbaceous, floral, spicy, phenolic, salty, and chemical). They scored their response to these characteristics according to a five-point hedonic scale depending on the intensity of the perceived stimulus for each attribute, with 0 indicating the absence of the stimulus and 4 indicating a strong stimulus (0 = absence, 1 = very low, 2 = weak, 3 = moderate, and 4 = strong).

### 2.7. Overall Yield and Kinetic Modeling of the Yeast Biomass, Substrate Consumption, and Ethanol Concentration

Once the judges indicated their preference for the product of the M2–23 versus M2–19 culture medium, fermentation was carried out with the former to determine the kinetics of biomass growth, ethanol production, and consumption of the reducing sugars. With the data, a determination was made of the yield of biomass growth in relation to the substrate consumption (*Y_X_*_/*S*_, g g^−1^) and the yield of the product with respect to the substrate consumption (*Y_P_*_/*S*_, g g^−1^), using Equations (4) and (5), respectively.

(4) $$Y_{X/S}=\frac{X_{t}-X_{0}}{S_{0}-S_{t}}$$

(5) $$Y_{P/S}=\frac{P_{t}-P_{0}}{S_{0}-S_{t}}$$

Furthermore, the data were fed into the Gompertz and Logistic models (Table 1) to identify the model that best matched the experimental data.

**Table 1:** Kinetic models.

Model	Equation	
Logistic model [36]	$Y=Y_{M}\frac{Y_{0}}{\left(Y_{M}-Y_{0}\right)\exp\left(-kt\right)+Y_{0}}$	(6)
Gompertz model, modified and re-parameterized by Norton [37]	$Y=Y_{M}{\left(\frac{Y_{0}}{Y_{M}}\right)}^{exp(-kt)}$	(7)

*Y* can represent the biomass growth (*X*), substrate consumed (*S*), or ethanol production (*P*), each in g L^−1^. *Y_0_* is the initial concentration of these variables (*X_0_*, *S_0_*, *P_0_*) calculated for the model in g L^−1^. *Y_M_* denotes the maximum values predicted by the model for the biomass growth (*X_M_*), ethanol concentration (*P_M_*), and consumption of the substrate (*S_M_*), expressed in g L^−1^. The values *t* and *k* (in days) denote the time of incubation and the time lag, respectively.

### 2.8. Analytical Methods

#### 2.8.1. Determinations Made to Monitor the Kinetics of Mead Production

At different fermentation times, samples of 2 mL were taken in aliquots from the cell suspension and filtered under vacuum through Whatman GF/A membranes (1.6 μm, Cytiva, St. Louis, MO, USA) at a constant weight. The concentration of the biomass was evaluated by the dry weight method, and the pH of the filtrate was measured with an Oakton OKT35613-54 potentiometer (Cole-Parmer, Vernon Hills, IL, USA) [38]. The amount of ethanol was ascertained with the microdiffusion method, employing a solution of K_2_Cr_2_O_7_ (JT Baker, Xalostoc, Mexico State, Mexico; purity >99%) as the oxidizing agent [39]. The enzymatic method with glucose oxidase and peroxidase was utilized for the glucose determination [40]. The reducing sugars were quantified by the of 3,5-dinitrosalicylic acid method (DNS) [41], and yeast assimilable nitrogen (YAN) was quantified by the formaldehyde method [42]. Total phenols were examined with the reagent Folin-Ciocalteu at 10%, as described previously [15], expressing the data as the mg L^−1^ gallic acid equivalent (GAE). Finally, the antioxidant capacity was appraised with the 2,2′-azino-bis(3-ethylbenzothiazoline-6-sulfonic acid) (ABTS) radical (Sigma Aldrich, Toluca, Mexico State, Mexico) and expressed in an mg L^−1^ Trolox equivalent (TE) of mead [15].

#### 2.8.2. Evaluation of the Physicochemical and Microbiological Characteristics of the Mead

The physicochemical properties of the final product were assessed by means of certain parameters reported in the literature, considering as a reference some of the norms of quality established for beer and wine. To our knowledge, there are no standardized norms for mead.

The percentage of alcohol was measured with an alcohol hydrometer at 20 °C (% *w*/*v*) [43]. The pH and the total amount of reducing sugars were determined as previously described. The density was evaluated by the pycnometer method [44], and the total acidity, the total sulfur dioxide, and the free sulfur dioxide were evaluated by volumetric titration [45,46]. The content of 5-hydroxymethylfurfural (HMF) (Sigma Aldrich, Toluca, Mexico State, Mexico) was evaluated by the dilution of the corresponding sample, making an HMF curve from an acetonitrile: water (50:50 *v*/*v*) solution with a concentration of 0 to 10 μg/mL, read on a spectrophotometer at 284 nm [47]. The aldehydes, esters, methanol, and higher alcohols were quantified by gas chromatography (Clarus 480, Perkin Elmer, Whatman, MA, USA) [48].

The content of lead and arsenic was assessed on an atomic absorption spectrometer (PinAAcle 900H, Perkin Elmer, Whatman, MA, USA) with a flow injection system (FIAS 100, Perkin Elmer, Whatman, MA, USA) [49]. The presence of coliform bacteria, mesophilic aerobic microorganisms, fungi, and yeasts was examined microbiologically [50,51,52] and quantified as colony forming units (CFUs).

#### 2.8.3. Analysis of Mid-Infrared Spectroscopy

Fourier transform mid-infrared spectroscopy (FTIR-MIR) was used to evidence the molecular bonds existing in the samples. The purpose of this analysis was to provide greater evidence of the changes that occur in the M2–23 medium during fermentation and to establish reference data for future studies on fermentation with *P. kudriavzevii*. The measurements were made on a Frontier spectrophotometer (PerkinElmer, Waltham, MA, USA) equipped with an attenuated total reflection (ATR) accessory with a SeZn crystal (Pike Technologies, Madison, WI, USA). The spectra were acquired in triplicate at 22 °C in the interval of 4000–700 cm^−1^ at a resolution of 4 cm^−1^, using 64 scans per analysis. Spectra were processed and compared by means of the Compare tool on Spectrum software version 10.5.3.738 (Perkin Elmer, Waltham, MA, USA).

### 2.9. Establishment of the Parameters of the Kinetic Models and Statistical Analysis

Non-linear kinetic modeling and the establishment of the parameters were achieved on GraphPad Prism version 10.0 (GraphPad Software, La Jolla, CA, USA). The current results are expressed as an average of at least four replicates for kinetic experiments and three experiments for the physicochemical analysis of honey. Significant differences were determined by two-way analysis of variance (ANOVA) and Tukey’s test (*p* < 0.05). The X^2^ statistical analysis was applied to the data from the sensory tests, using the minimum number of correct judgements to reach significance [15,53].

## 3. Results and Discussion

### 3.1. Evaluation of Fermentation in Different Culture Media

**Figure 1:**
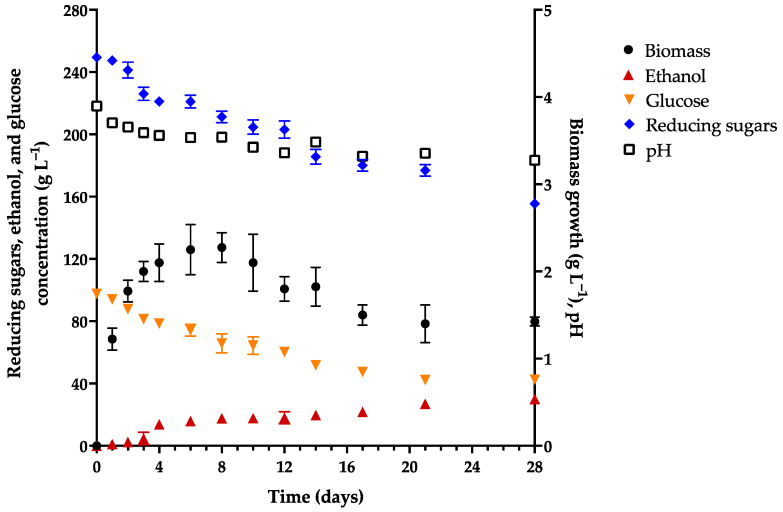
Kinetics of the pH, yeast biomass growth, and the concentration of ethanol, glucose, and reducing sugars in the control medium (M0) containing honey at 23 °Bx (317 g L^−1^) and *Pichia kudriavzevii* 4A as the starter culture.

The M0 medium, a solution of honey, was employed to examine the need to add salts and organic growth factors for the production of mead in the presence of *P. kudriavzevii* 4A as the starter culture. The data on pH, yeast biomass growth, and the concentration of ethanol, glucose, and reducing sugars are shown in Figure 1.

As can be appreciated in Figure 1, there was no phase lag during the yeast growth, which is because the culture had previously adapted itself to a medium with honey (M0). The maximum growth of yeast cells occurred from day 6 to day 8 of incubation, reaching a concentration of 2.26 g L^−1^. Subsequently, the biomass began to decline despite the presence of a sufficient substrate for growth and the continued use of the substrate by *P. kudriavzevii* 4A. By day 10 of fermentation, the yeast had only consumed 17.9% of the initial quantity of reducing sugars and 34% of the initial amount of glucose in the medium. By the end of 28 days, the yeast had only consumed 56.4% of the total glucose and 37.7% of the reducing sugars.

The concentration of ethanol was notable as of day 4, reaching its maximum level on day 21 and then remaining at the same level without any significant change until day 28 (3.8% *v*/*v*, 29.96 g L^−1^). This level of ethanol is well below the minimum that mead should contain (8% *v*/*v*) [5,6]. A possible reason for the behavior of the yeast is the low pH of the M0 medium, which began at 3.9 and declined to 3.28 by day 12.

With a low pH (<3.0), *S. cerevisiae* requires more time to generate ethanol, generates a limited quantity, has a low consumption of sugars in the medium, and affords a final product with high acidity [54]. *P. kudriavzevii* is known for its capacity to grow at a lower pH than *S. cerevisiae*, even at pH values as low as 1.5. However, it reportedly grows better at an initial pH of 3 to 6 [55,56]. Many studies that have explored the capacity of *P. kudriavzevii* to produce ethanol or other higher alcohols in beverages have experimented with an initial pH of approximately 5.0 for the culture medium [27,28], finding a final pH of about 3.6 [32,56].

Other possible reasons for the limited production of ethanol and yeast growth in the M0 medium could be the formation of 5-hydroxymethylfurfural (HMF) during fermentation and the scarce assimilable nitrogen (YAN) available in the medium. Regarding the latter factor, honey has a very low content of proteins and therefore of total nitrogen [3]. The values found for these compounds in M0 before and after fermentation are shown in Table 2, along with additional characteristics.

At high concentrations, 5-HMF damages the cellular structure of yeasts and thus reduces their growth. 5-HMF is formed by the dehydration of fructose in honey, a process promoted by a pH of the medium of less than 3.0 and a temperature of fermentation greater than 25 °C. Additionally, it inhibits enzymatic activity, which is important for the synthesis of ethanol [15,57]. However, the concentration of 5-HMF in the culture medium (~44 mg L^−1^, Table 2) was close to that reported for commercial mead (46–280 mg L^−1^) [15]. Therefore, this compound was not responsible for inhibiting the formation of biomass or ethanol.

In a medium used for yeast maintenance and reproduction, assimilable nitrogen is an essential nutrient for the synthesis of critical enzymes, structural proteins, nucleic acids, and ribonucleotides. According to diverse studies, many yeasts require at least 267 mg L^−1^ of YAN for the efficient conversion of sugars to ethanol [58] and between 100 and 300 mg L^−1^ for the generation of esters and higher alcohols that confer agreeable fruity and floral flavors and aromas to the fermented beverage [20]. The initial concentration of YAN in the M0 medium (8.75 mg L^−1^), far below the suggested level, was completely consumed by the yeast. Hence, the limited growth of *P. kudriavzevii* 4A and the limited production of ethanol were probably mainly caused by the low level of YAN and a low pH.

**Figure 2:**
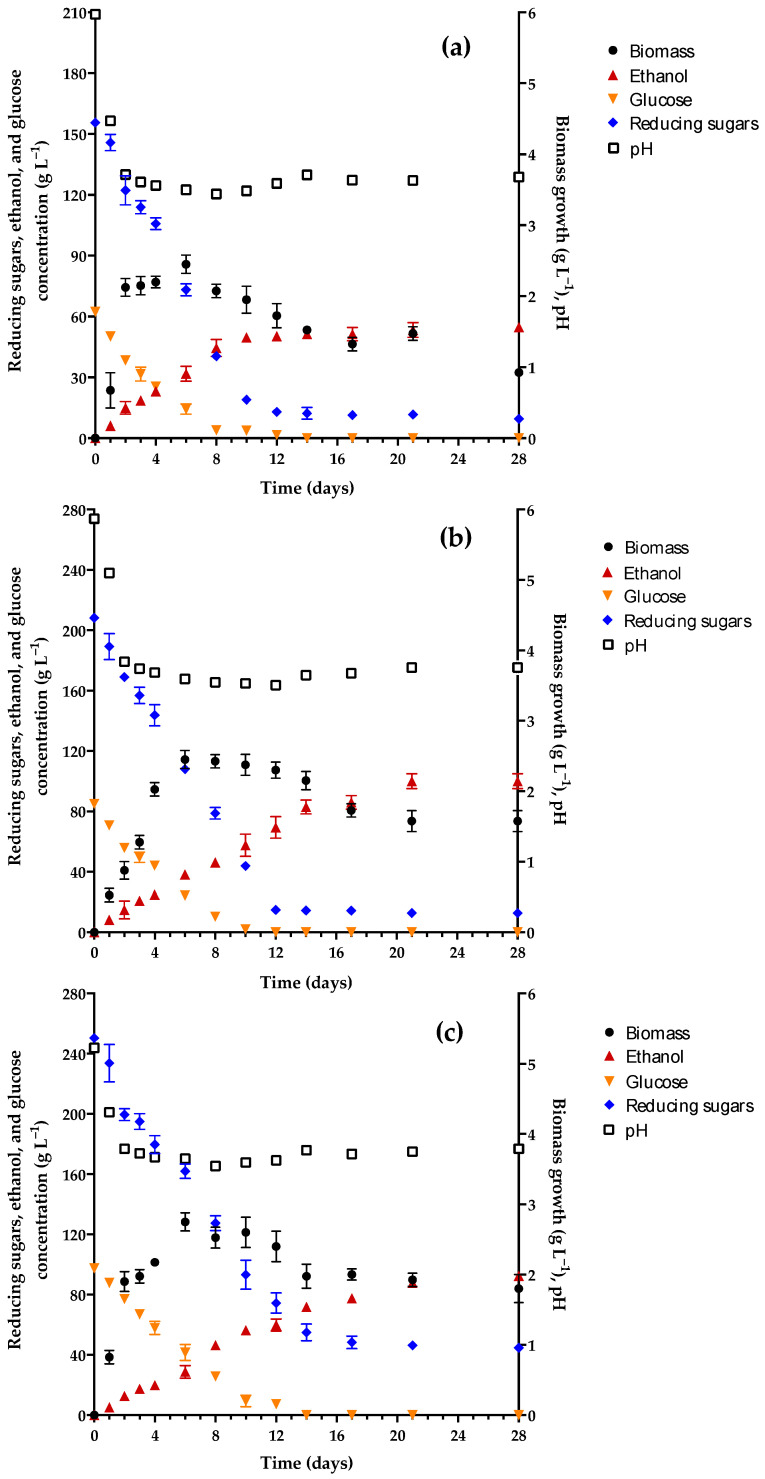
Kinetics of the pH, yeast biomass growth, and concentration of ethanol, glucose, and reducing sugars in the enriched medium (M1) containing honey at (**a**) 15, (**b**) 19, or (**c**) 23 °Bx (equivalent to 203, 256, and 317 g L^−1^, respectively) and *Pichia kudriavzevii* 4A as the starter culture.

Based on the results, M0 was reformulated to obtain an improved medium. Ammonium sulfate was included because it has been demonstrated to be a good source of nitrogen for *P. kudriavzevii*. Contrarily, nitrates and nitrites are not assimilated by this yeast [32,55]. Other salts were placed in the medium to provide minerals and nutrients. Monobasic potassium phosphate was added as a source of phosphorous, which is essential for the synthesis of nucleic acids and phospholipids [59]. Yeast extract was added as a rich source of nutrients because it is proven to increase the production of ethanol by *P. kudriavzevii* [60]. Compared to M0, this enriched medium (M1) exhibited an improved kinetic of pH, biomass growth, and concentration of reducing sugars, glucose, and ethanol at each of the three concentrations of honey tested (Figure 2).

Table 3 illustrates the values of some physicochemical parameters of the M1 medium before and after fermentation.

Compared to M0, M1 (at all three concentrations of honey) showed much higher initial values of pH (5.23 ≤ pH ≤ 5.97 vs. 3.9) and YAN (553 mg L^−1^ ≤ YAN ≤ 584.5 mg L^−1^ vs. 8.75 mg L^−1^) as a consequence of the salts added. These modifications favored the generation of ethanol, which was evident as of the first day.

The concentration of ethanol in M1–15 was 6.50% *v*/*v* (51.32 g L^−1^) on day 14 of fermentation. In relation to this value, the 7.14% *v*/*v* (56.37 g L^−1^) found on day 28 did not represent any significant change. Although the final product had a greater percentage of ethanol than M0 (7.14% vs. 3.8% *v*/*v*), it still cannot be considered mead, a beverage with a minimum of 8% ethanol. Contrarily, the products of the M1–19 and M1–23 media both reached their maximum concentration of ethanol at 21 days of fermentation, being 12.67% *v*/*v* and 11.12% (99.96 g L^−1^ and 87.73 g L^−1^), respectively. These values were not significantly different on day 28.

*P. kudriavzevii* 4A consumed the substrate more efficiently in the M1 versus M0 medium. The glucose in the medium was totally exhausted at around day 14 of fermentation. Regarding the reducing sugars, 92.3% were consumed as of day 12 in M1–15 and M1–19 and 80.6% were consumed as of day 14 in M1–23. In each case, there was no significant decrease in the concentration of reducing sugars for the remainder of the experiment (up to day 28). Although the composition of honey can vary in accordance with its origin, its main reducing sugars are glucose and fructose, followed by a lesser quantity of maltose [61]. Whereas glucose and fructose are easily assimilated and utilized by *P. kudriavzevii* as substrates to produce ethanol [59,62], the use of maltose may be less efficient [32], possibly explaining the incomplete consumption of reducing sugars.

The maximum level of yeast biomass was observed on day 6 of fermentation for all three types of M1. The generation of the biomass of *P. kudriavzevii* 4A was the same for M1–15 and M1–19 (2.45 g L^−1^) and very similar for M1–23 (2.75 g L^−1^). After day 6, the biomass began to decline (as found with M0), coinciding with the consumption of 90% of the glucose in the medium and the minimum pH registered (~3.5). As can be appreciated, the pH has a great influence on the growth of *P. kudriavzevii* 4A.

Other important results of the final product are shown in Table 3. The titratable acidity (2.32–3.11 g L^−1^) and the concentration of 5-HMF (37.57–52.21 mg L^−1^) were within the range of reported values (2.2–7.08 g L^−1^ and 46–280 mg L^−1^, respectively) [15,63]. The process of fermentation caused the initial concentration of YAN to diminish by about 9% (~53 mg L^−1^), leaving a large quantity of assimilable nitrogen in the medium (498.75–533.75 mg L^−1^). Supplementing the medium with assimilable nitrogen promotes the formation of medium-chain fatty acid (MCFA) ethyl esters, which contribute to fruity flavors in the beverage [64]. On the other hand, if the nitrogen concentration is over 480 mg L^−1^, there may be a higher level of ethanol and acetic acid in the mead, leading to a decrease in the perception of a fruity flavor and a greater aroma and flavor similar to nail polish remover, as described by Torrea et al. [64] in wine and by Pereira et al. [58] in mead. Accordingly, the beverages derived from M1–19 and M1–23 had flavors uncharacteristic of mead, being bitter and astringent with hints of unpleasant acidity.

**Figure 3:**
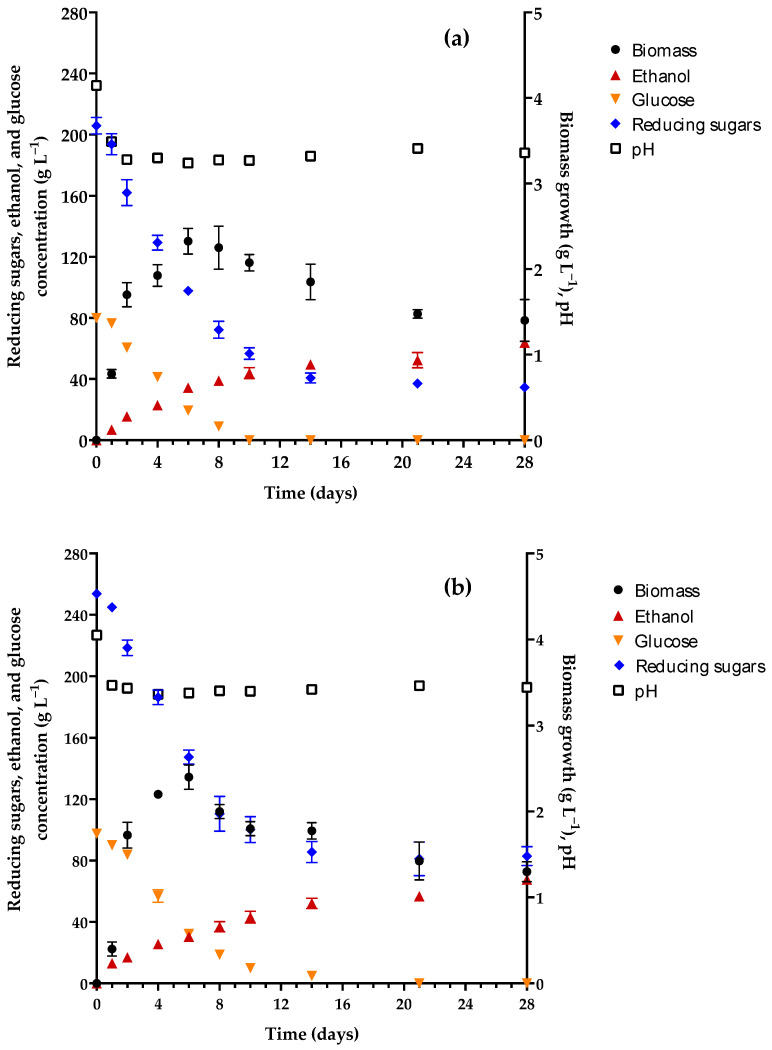
Kinetics of the pH, yeast biomass growth, and concentration of ethanol, glucose, and reducing sugars in the second enriched medium (M2) containing honey at (**a**) 19 and (**b**) 23 °Bx (256 and 317 g L^−1^, respectively) and *Pichia kudriavzevii* 4A as the starter culture.

Due to the unpleasant taste of the two products with an acceptable ethanol content (obtained from M1–19 and M1–23), the culture medium was again reformulated. The new formulation (M2) contained a lower quantity of ammonium sulfate and monobasic potassium phosphate and the same concentration of yeast extract. Some of the salts used in M1 were omitted from M2, which was tested with only two concentrations of honey (19 and 23 °Bx). The kinetics of M2–19 and M2–23 are illustrated in Figure 3a,b, respectively. The characteristics of the M2 medium before and after fermentation are shown in Table 4.

The maximum growth of *P. kudriavzevii* 4A in M2–19 and M2–23 was approximately 2.36 g L^−1^, with no significant difference between the two media. Thus, the level of yeast biomass in M2 was slightly lower than the 2.45–2.75 g L^−1^ found in M1. The biomass in M2 reached its maximum level on day 6 (as found in M1). The posterior decline in biomass coincided with the minimum pH registered (3.26 and 3.37 for M2–19 and M2–23, respectively). The initial pH of the M2 media was 4.1, higher than the 3.9 pH of M0 but lower than the range of M1 (5.23 ≤ pH ≤ 5.97), apparently due to the omission of some salts present in the latter.

Since the concentration of 5-HMF in M2 (47.48–57.68) was within the acceptable interval for mead (46–280 mg L^−1^) [15], this parameter did not seem to interfere with the generation of yeast biomass.

As there was less ammonium sulfate in M2 than in M1, the initial value of YAN was lower. The ~200 mg L^−1^ found in M2 (versus ~570 mg L^−1^ for M1) corresponds to the moderate range (160–320 mg L^−1^), which lends itself to the attainment of adequate physicochemical and sensory properties [64]. The assimilable nitrogen consumed by the yeast in M2 was approximately 46 mg L^−1^, lower than the 53 mg L^−1^ consumed in M1. Therefore, *P. kudriavzevii* 4A consumed 23% of the YAN in M2, lower than the utilization of nitrogen reported for the production of wine and mead with *S. cerevisiae* (>86%) [20,58,64]. Furthermore, glucose was totally consumed, and reducing sugars were partially consumed (82% for M2–19 and 67.2% for M2–23). The concentration of ethanol reached 8.04 and 8.57% *v*/*v* on day 28 of fermentation with M2–19 and M2–23, respectively, thus reaching a sufficient level to qualify as mead.

When comparing the data between the M0, M1, and M2 media, it can be appreciated that the supplementation with ammonium salt to provide nitrogen in the media favored the generation of ethanol by the yeast as of the first day of fermentation. Importantly, the greatest quantity of ammonium sulfate assayed (being in M1) led to the beverage with the greatest percentage of ethanol (12.65%), produced in only 21 days. However, M2 afforded a beverage with a more pleasant appearance, aroma, and flavor.

### 3.2. Sensory Test of the Final Products Obtained with M2

The meads derived from M2–19 and M2–23 were compared in a sensory test, resulting in a clear preference of the judges for the latter (73.33% vs. 26.67%; Figure S1, Supplementary Material). The judges commented that the former beverage had a balance between sweetness and alcohol and a very pleasant aroma of flowers (orange blossom) and fruit (pear and apple). Contrarily, M2–19 was described by some panelists as having an unpleasant contrast between a sweet and attractive aroma and a taste of an elevated level of alcohol.

Curiously, the percentage of ethanol in both samples was similar, being slightly higher in M2–23. Thus, the taste of an elevated level of alcohol in M2–19 was based on a much lower concentration of residual reducing sugars in this medium compared to that in the M2–23 medium (34.55 versus 82.91 g L^−1^). In mead, residual reducing sugars in a range of 70.81–160 g L^−1^ commonly furnish a pleasant sweet taste [9], which conjugates favorably with the aroma and percentage of alcohol. Hence, the subsequent evaluations were carried out with the product of the M2–23 medium.

### 3.3. Comparison of the Sensorial Attributes of the Mead from M2–23 with Those of a Commercial Sample

**Figure 4:**
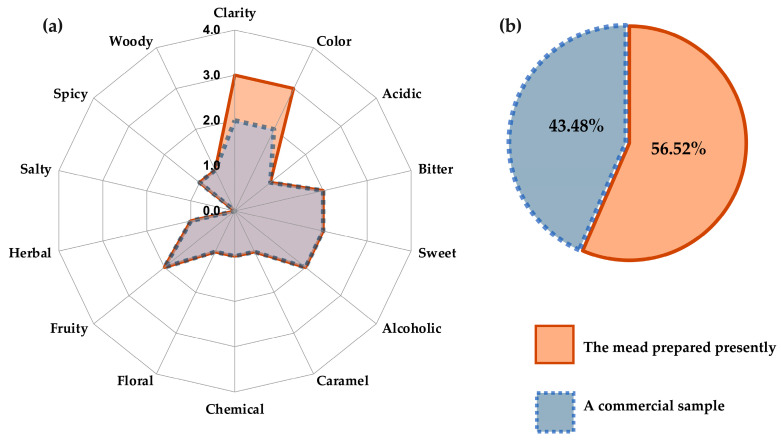
Sensory profile test (**a**) and the overall preference (**b**) of a panel of judges, comparing the present mead (prepared in the M2–23 medium with *Pichia kudriavzevii* 4A) to a commercial sample (elaborated with *S. cerevisiae*).

The attributes of the mead derived from M2–23 were compared with those of a commercial sample (Figure 4a). The overall preference of the panel of judges is portrayed in Figure 4b. As can be appreciated, the mead elaborated with *P. kudriavzevii* 4A complies with the aim of the current contribution of creating a promising alternative for the elaboration of a mead that will likely be accepted by consumers. Moreover, the process of production was faster than the traditional process based on a *Saccharomyces* yeast strain, the latter of which takes up to 6 months [65].

Three attributes were evaluated: appearance (color and clarity), flavor (sweet, acidic, and bitter), and aroma (fruity, woody, alcoholic, herbal, floral, spicy, caramel, salty, and chemical). As can be appreciated in Figure 4a, the only difference between the mead produced in this study and the commercial sample is in relation to appearance. The mead elaborated with *P. kudriavzevii* 4A was considered more attractive because of its color and clarity. Both beverages were perceived as balanced between a bitter and sweet taste. Although several pleasant nuances were perceived in both, the flavor was qualified as mainly fruity and alcoholic. The preference of the judges (Figure 4b) slightly favored the mead presently produced. According to the χ^2^ statistical analysis, however, there was no significant difference in preference (0.5434 < 3.841), in part owing to the number of judges [53].

The mead herein elaborated in 28 days turned out to have a consumer acceptance equivalent to the commercial variety, which usually takes 3–6 months to prepare with *S. cerevisiae* [65]. Thus, the current non-*Saccharomyces* starter culture may furnish an economic advantage over commercial yeasts in the manufacturing of mead while at the same time providing a safe, visually pleasant, and good-tasting product.

### 3.4. The Kinetic Parameters Used to Characterize the Mead Derived from M2–23

**Figure 5:**
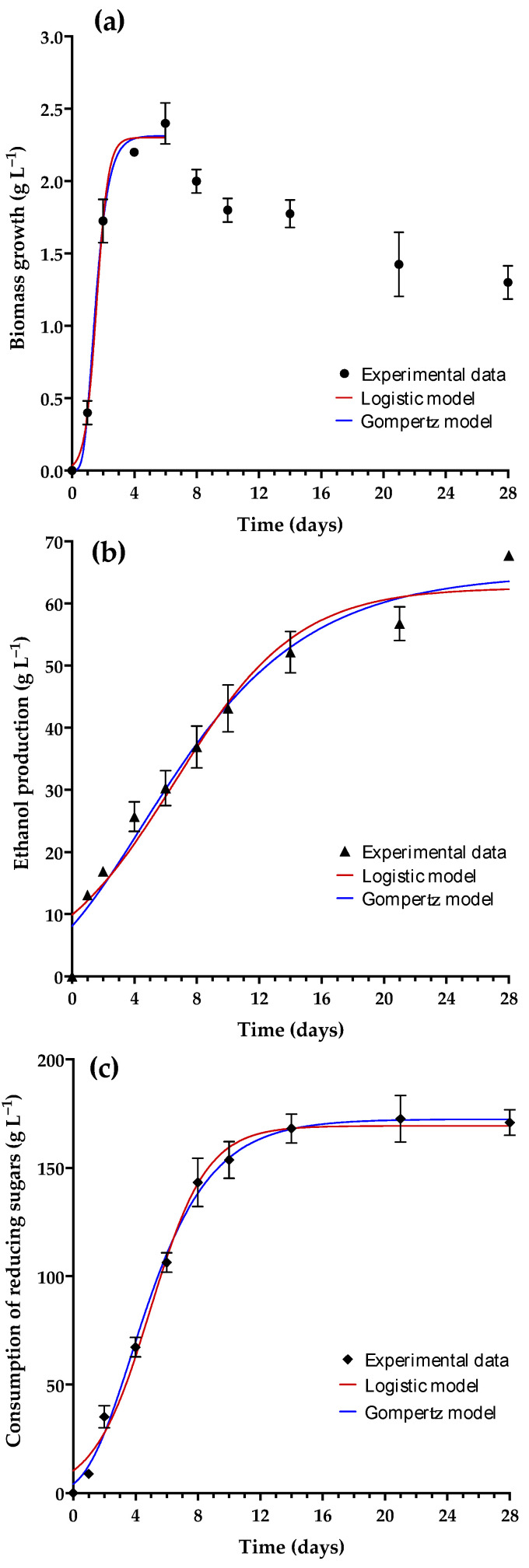
Kinetic models of the (**a**) yeast biomass growth, (**b**) ethanol production, and (**c**) consumption of reducing sugars during the elaboration of mead from the M2 medium with 23 °Bx of honey (M2–23) and *Pichia kudriavzevii* 4A as the starter culture.

The kinetics of biomass growth, ethanol production, and consumption of reducing sugars during fermentation in the M2–23 medium (Figure 5) were fed into the Logistic and Gompertz models (see Table 1) in order to be able to predict the parameters at any given fermentation time (Table 5). Additionally, the *Y_X_*_/*S*_ and *Y_P_*_/*S*_ yields were obtained and are shown in Figure S2 (Supplementary Material).

To evaluate the yeast biomass growth (Figure 5a), modeling was carried out until the beginning of the stationary phase (at day 6), at which time the yeast growth declined. The experimental growth of the biomass and the quantity of sugars consumed (Figure 5c) are well predicted by both the Logistic and Gompertz models. The experimental data on the ethanol concentration (Figure 5b) are also very near to the values predicted by the models, except that the models indicate a concentration of about 10 g L^−1^ of ethanol at time zero when the real concentration is zero.

Although the kinetic parameters of both models are similar, the Gompertz model is best for describing the experimental data. The predictions of this model (*X_M_* = 2.31 g L^−1^, *P_M_* = 64.83 g L^−1^, *S_M_* = 172.40 g L^−1^) are very close to the maximum level of the biomass (*X_Mexp_* = 2.40 g L^−1^) and ethanol (*P_Mexp_* = 67.75 g L^−1^) and the maximum consumption of the substrate (*S_Mexp_* = 172.58 g L^−1^) found experimentally. In each of these three cases, the very short lag time given by the model coincides with the experimental observations.

The highest *Y_X_*_/*S*_ values occurred at 24 and 48 h (approximately 0.049 g g^−1^), which coincides with a noticeable fermentation growth stage. The maximum biomass yield found on day 6 resulted in a *Y_X_*_/*S*_ value of 0.023 ± 0.002 g g^−1^. Subsequently, the growth of the yeast decreased, as did the biomass yield values. Thus, the lowest value of *Y_X_*_/*S*_ (0.008 ± 0.001 g g^−1^) was observed on day 28. On the other hand, the ethanol yield (*Y_P_*_/*S*_) showed its highest value (1.525 ± 0.332 g g^−1^) after 24 h of fermentation and then progressively decreased over time, reaching 0.397 ± 0.013 g g^−1^ at day 28.

To the best of our knowledge, there are no reported yield values for mead production with *P. kudriavzevii*, so it was not possible to compare the present data with other studies with this yeast. However, it has been observed that the yields given by *S. cerevisiae* depend on the variety of the yeast as well as the conditions and time of cultivation. García et al. [66] studied *S. cerevisiae* for 55 h of fermentation and obtained a final biomass production of 2.3 g L^−1^, a substrate consumption of 138.96 g L^−1^, and an ethanol production of 6.5 g L^−1^, resulting in values of 0.017 g g^−1^ for *Y_X_*_/*S*_ and 0.047 g g^−1^ for *Y_P_*_/*S*_. These values are lower than those obtained for *P. kudriavzevii* 4A after 48 h of culture (*Y_X_*_/*S*_ = 0.05 g g^−1^ and *Y_P_*_/*S*_ = 0.49 g g^−1^). On the other hand, when using *S. cerevisiae* subsp. *bayanus* and *S. cerevisiae* IM8 and JP14, the *Y_P_*_/*S*_ values were between 0.344 and 0.52 g g^−1^ at 20–28 days of culture [67,68], close to the 0.397 g g^−1^ value obtained with *P. kudriavzevii* 4A after 28 days of fermentation.

### 3.5. Kinetics of Antioxidants during Fermentation

**Figure 6:**
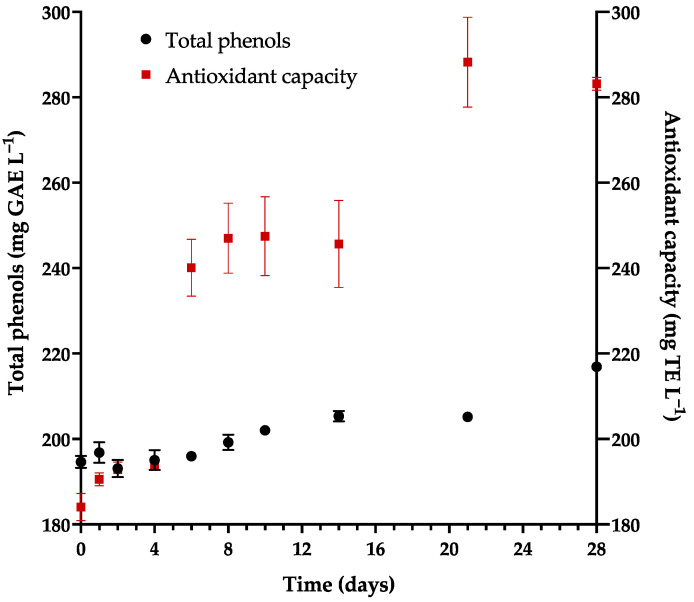
Phenolic compounds and antioxidant capacity during fermentation in the M2–23 medium.

In the M2–23 medium, the initial values were 194.62 mg GAE L^−1^ for the concentration of total phenols and 184.07 mg TE L^−1^ for the antioxidant capacity (measured with ABTS). During the 28 days of fermentation, there was an 11.4% increase in phenolic compounds and a 53.82% increase in antioxidant capacity (Figure 6).

The few reports that mention the change in the level of phenolic compounds and antioxidant capacity of honey mead during fermentation provide clear evidence of an increase in these parameters over time [15,69]. In the mead derived from M2–23, the values of total phenols (216.9 mg GAE L^−1^) and the antioxidant capacity (1.13 mM TE L^−1^, 283.15 mg TE L^−1^) are close to those found for a mead elaborated in Poland (236.68 mg GAE L^−1^; 1.39 mM TE L^−1^; 347 mg TE L^−1^) [15].

It is documented that the addition of some fruits and herbs affords a significant increase in the antioxidant capacity of mead [70]. On the other hand, phenolic compounds are closely related to the origin of honey and the ingredients involved in its production. Mead made with clear monofloral honey is reported to have a lower content of total phenols (210.5 mg GAE L^−1^) and thus a lower antioxidant capacity (1.8 mM TE L^−1^) [71] than mead prepared from dark multifloral honey with red fruit added. In the latter case, the values in the literature range from 2.04 to 4.13 mM TE L^−1^ [6,71,72].

### 3.6. Characteristics of Mead Derived from the M2–23 Medium

Physicochemical and microbiological parameters were determined for the mead elaborated in the M2–23 medium with *P. kudriavzevii* 4A (Table 6). The results are compared to the norms for wine and the values described in some studies for mead. To our knowledge, there is currently no norm for mead.

**Table 6:** Characteristics of the mead derived from the M2–23 medium (with *Pichia kudriavzevii* 4A).

Characteristic	Result	Specification/Reported Range	Reference
pH	3.51 ± 0.015	2.49–4.2	[73,74,75,76]
Density (g cm^−3^)	1.012 ± 0.001	0.9757–1.293	[73,77]
Ethanol (% *v*/*v*)	8.57 ± 0.03	8–18	[8,25,42]
Methanol (mg 100 mL^−1^ AA)	<DL	<300	[78]
Higher alcohols (mg 100 mL^−1^ AA)	122 ± 0.20	58–129	[25,73]
Aldehydes (mg 100 mL^−1^ AA)	13.46 ± 0.11	6.2–125.5	[63,73]
Esters (mg 100 mL^−1^ AA)	332.09 ± 0.23	24.72–317	[8,79]
Total acidity (g TA L^−1^)	2.812 ± 0.14	2.2–7.7	[42,63,73,76]
Total sulfur dioxide (mg L^−1^)	6.58 ± 0.02	5–275	[76]
Free sulfur dioxide (mg L^−1^)	5.35 ± 0.05	1–61	[20,76]
Reducing sugars (g L^−1^)	82.91 ± 6.16	> 50	[78]
Antioxidant capacity (mM TE L^−1^)	1.13 ± 0.005	1.39–4.13	[15,71,72]
Total phenols (mg GAE L^−1^)	216.9 ± 0.51	210.5–303.2	[15,71]
Lead (mg L^−1^)	<LQ	<0.5	[78]
Arsenic (mg L^−1^)	<LQ	<0.5	[78]
Fungi (CFUs mL^−1^)	Absent	Absent	[51]
Yeast (CFUs mL^−1^)	Absent	Absent	[51]
Total coliform bacteria (CFUs mL^−1^)	Absent	Absent	[52]
Aerobic mesophiles (CFUs mL^−1^)	Absent	Absent	[50]

AA, anhydrous alcohol; TA, tartaric acid; TE, trolox equivalent; GAE, gallic acid equivalent; CFUs, colony forming units; DL, detection limit for methanol (12.6 mg 100 mL^−1^ AA); LQ, limit of quantification (for lead, 0.30 mg L^−1^; for arsenic, 0.002 mg L^−1^).

The pH and density of the mead prepared with *P. kudriavzevii* were within the range of values described in the literature [73,76,77]. The ethanol content of 8.57 ± 0.03% *v*/*v* complied with the requirement for mead (8–18% *v*/*v*) [5]. The innocuousness of consuming the final product is evidenced by the absence of detectable methanol, lead, arsenic, and pathogenic microorganisms (Table 6). Gas chromatography revealed the presence of acetaldehyde, ethyl acetate, 2-pentanol, and isoamyl alcohol.

The concentrations of aldehydes (13.46 mg 100 mL^−1^ AA) and esters (332.09 mg 100 mL^−1^ AA) in the beverage were close to the values reported in the literature for meads [63,73,79]. Acetaldehyde, in low concentrations, is known to confer a fresh flavor of green leaves, while fruity and floral aromas are attributed to the ester (ethyl acetate) in the beverage. The two higher alcohols identified in the product, 2-pentanol and isoamyl alcohol, provide an aroma of a fermented alcoholic beverage similar to that of a sweet white wine with nuances of mature bananas and yellow apples [25,42,79].

The acidity was greater in the final product than in the initial medium (measured as tartaric acid (TA): 2.81 ± 0.14 g L^−1^ versus 1.16 ± 0.08 g L^−1^, respectively) due to the high concentration of acids generated during fermentation. Pereira et al. [42] described an acidity of 5 g TA L^−1^ in mead elaborated with *S. cerevisiae* immobilized in alginate. Morales et al. [73] reported 2.90 g L^−1^ of TA in mead after fermentation.

The current values for free and total sulfur bioxide (5.35 mg L^−1^ and 6.58 mg L^−1^, respectively) were within the range of the values observed in other studies [76]. This compound, derived from the oxidation and growth of some non-*Saccharomyces* yeasts, avoids the contamination of the product. However, at high concentrations, it confers unpleasant flavors to the beverage, such as that of white pepper and soil. At concentrations above 100 mg L^−1^, sulfur bioxide has been proven to inhibit the generation of ethanol and the consumption of sugars [76,80].

As aforementioned, it is desirable for the final product to contain sugars because they afford a sweet flavor that can provide a balance with the flavor of alcohol. Sweet meads have a greater amount of residual sugars (21.4 to 117.2 g L^−1^) than dry meads (0.2 to 20.3 g L^−1^) [76], which in turn have a lower quantity than the norm for wine (>50 g L^−1^) [78]. Up to 199.6 g L^−1^ of residual sugars have been detected in meads [65]. The mead herein prepared, with 82.91 ± 6.16 g L^−1^ of residual sugars, is classified as sweet.

### 3.7. FTIR-MIR Spectra

**Figure 7:**
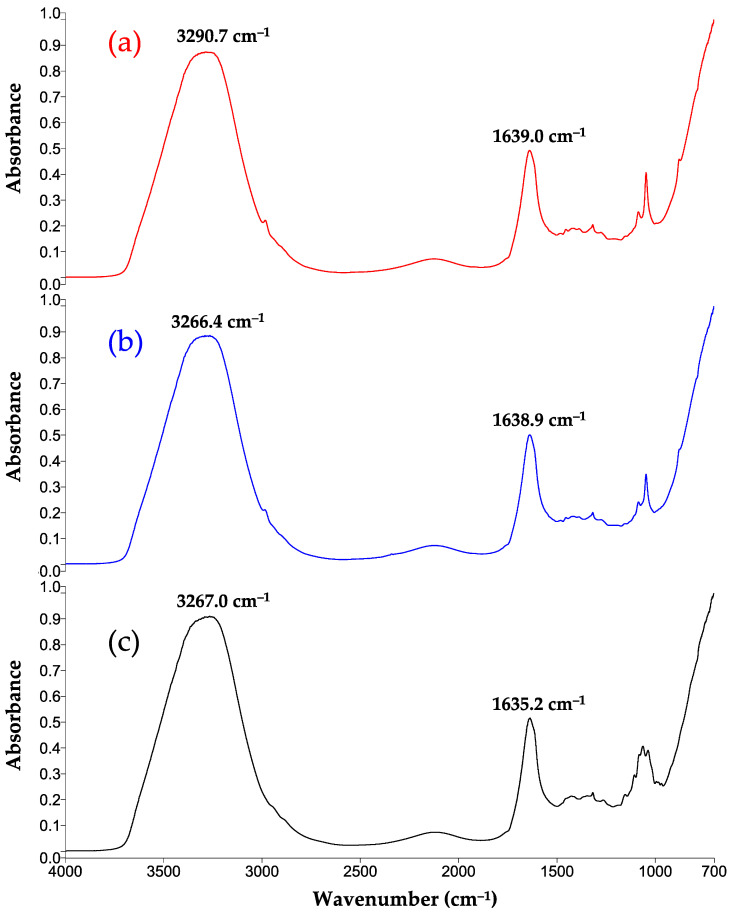
FTIR-MIR spectra in the region of 4000 to 700 cm^−1^ of (**a**) the mead herein elaborated with *Pichia kudriavzevii*, (**b**) a commercial mead made with *Saccharomyces cerevisiae*, and (**c**) the M2–23 medium before inoculation.

FTIR-MIR spectra for the present mead (Figure 7a), a commercial mead (Figure 7b), and the M2–23 culture medium before being inoculated (Figure 7c) show the type and origin of the absorption bands (Figure 7; data described in Table 7). The bands at 3269 cm^−1^ and 1638 cm^−1^ correspond to the stretching and deformation vibrations, respectively, of the hydroxyl group (-OH) in the aqueous medium and of ethanol formed during the fermentation of mead. The spectral region of 1200–900 cm^−1^ has absorption bands related to the bonding vibrations of carbohydrates (fructose, glucose, and maltose) and alcohols, which tend to overlap. The spectra of the current mead and the commercial sample show a percentage of similarity of 0.9919 based on their similar composition.

**Table 7:** Assignment of the bands for functional groups existing in the samples.

Functional Group	Origin of the Vibration	Position of the Bands ^a^ (cm^−1^)		
		M2–23 before Inoculation	The Present Mead	A Commercial Mead
O-H ν	Ethanol and water	3267	3290.7	3266.4
C-H ν	Alkyl groups (-CH_3_, -CH_2_)	-	2984.4	2986.3
C=O ν O-H δ	Organic acids Ethanol and water	1635.2	1639.0	1638.9
-O-CH_2_- δ	Esters	-	1453.5	1454.4
	Aldehydes	1424.4	-	-
O-H δ	In the C-OH group	-	1418.9	1419.3
C-H δ	Alkenes	1316.0	1316.2	1316.3
C-H ν C-O ν	Carbohydrates organic acids	1261.7	1274	1272
-C–O–C- ν	Carbohydrates such as glucose and fructose	1152	1153	1151
C–OH ν	Carbohydrates Organic acids	1103.9	-	-
CH-OH ν	Carbohydrates Ethanol	1080.6	1083.8	1083.7
CH_2_–OH ν	Carbohydrates	1060.7	-	-
C-OH δ	Ethanol	-	1044.5	1044.8
		1034.9	-	-
α-pyranose ring *sym*	Carbohydrates	993.2	-	-
C-OH ν	Carbohydrates	920	-	-
C-C ν	Disaccharides	899.1	-	-
-CH_3_ δ	Ethanol		877.5	878.7
C-H δ	Carbohydrates	867.1	-	-
		819.1	816.3	-
α-pyranose ring	Disaccharides	779.7	780.4	782.8

ν, stretching vibration; δ, deformation vibration; sym, symmetric vibration. ^a^ The interpretation of the bands is in accordance with Socrates [81], Cuenca et al. [82], and Kędzierska-Matysek et al. [83].

**Figure 8:**
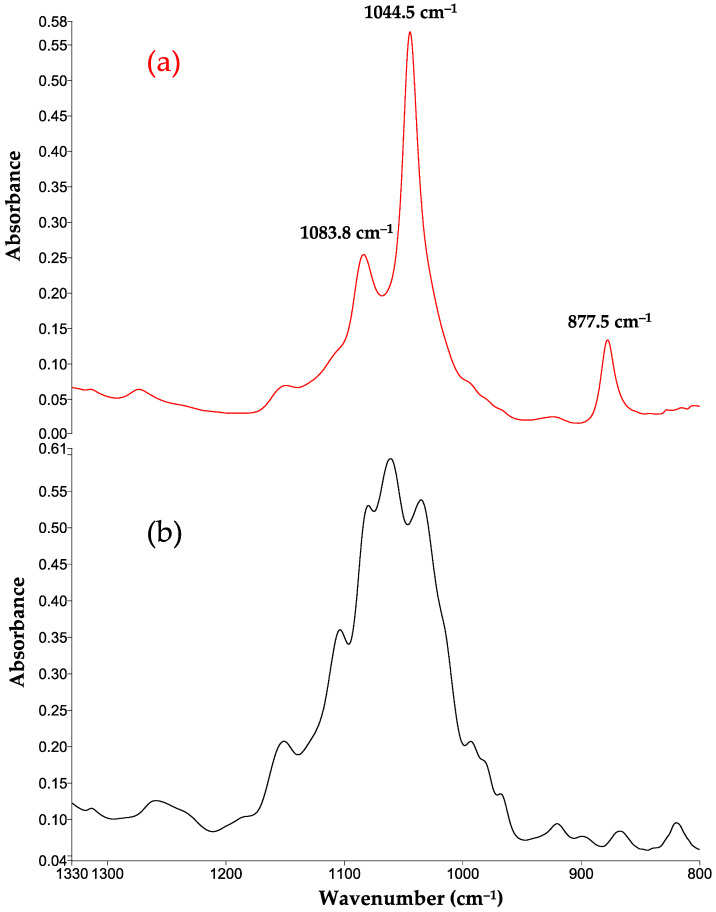
FTIR-MIR spectra in the region of 1330 to 800 cm^−1^ corresponding to (**a**) the mead prepared from the M2–23 medium and (**b**) the M2–23 medium before inoculation. HPLC water was used to provide the background spectrum.

To better understand the FTIR-MIR spectra of the present mead derived from M2–23 (Figure 8a) and the M2–23 medium before inoculation (Figure 8b), the two are illustrated at the interval of 1330 to 800 cm^−1^, finding a change in the bands of carbohydrates in the mead. Due to the presence of some unfermentable sugars, not all the bands corresponding to sugars disappear in the spectrum of the final product. Bands appear at 1083 cm^−1^ and 1044 cm^−1^, attributed to the alcohols (i.e., ethanol and isoamyl alcohol) generated by the consumption of sugars (i.e., glucose and fructose). The band at 877 cm^−1^ is principally related to the production of ethanol. These changes coincide with the reports by Cuenca et al. [82] and Fayolle et al. [84].

## 4. Conclusions

The current findings show that *P. kudriavzevii* 4A provides a suitable monoculture for producing mead when using a medium with 23 °Bx honey supplemented with ammonium sulfate (1.0 g L^−1^), monobasic potassium phosphate (0.5 g L^−1^), and yeast extract (1.0 g L^−1^). The presence of an appropriate level of YAN (about 200 mg L^−1^) and a moderate level of pH in the medium (in this case, within a range of 3.5–5.0) turned out to be crucial factors for the growth of *P. kudriavzevii* 4A as well as its consumption of sugars and generation of ethanol. The final product, obtained in 28 days, was a palate-pleasing mead with a pleasant aroma, an ethanol concentration greater than 8%, and a level of residual sugars that favors a balance between a sweet and alcohol flavor. Its floral and fruity nuances gave a favorable result in the overall preference test. The judges expressed a slightly greater preference (although not statistically significant) for the mead currently elaborated with *P. kudriavzevii* 4A compared to a commercial mead made with *S. cerevisiae*. The analyses of the quality of the final product showed that it is safe for consumption. Therefore, *P. kudriavzevii* 4A can be used to produce a safe mead with probable consumer acceptance in a relatively short period of time. Unlike artisanal mead, the current fermentation process was controlled, and the factors that affect the quality of the product are well defined.

## Figures and Tables

**Table 2:** Initial characteristics of the M0 medium (with *Pichia kudriavzevii* 4A as the starter culture) and of the final product.

Characteristic	Initial	Final
Ethanol (% *v*/*v*)	0	3.80 ± 0.08
Yeast assimilable nitrogen (mg YAN L^−1^)	8.75 ± 3.50	N.D.
Titratable acidity (g TA L^−1^)	0.53 ± 0.09 ^a^	2.03 ± 0.29 ^b^
5-HMF (mg HMF L^−1^)	46.66 ± 0.95 ^a^	43.94 ± 0.57 ^b^

N.D., not detected; TA, tartaric acid; HMF, hydroxymethylfurfural. The letters denote a significant difference for the corresponding characteristic (Student’s *t*-test, α = 0.05).

**Table 3:** The initial characteristics of the three types of M1 medium (with *Pichia kudriavzevii* 4A as the starter culture) and the corresponding final products.

Characteristic	M1–15		M1–19		M1–23	
	Initial	Final	Initial	Final	Initial	Final
Ethanol (% *v*/*v*)	0	7.14 ± 0.50 ^A^	0	12.67 ± 0.62 ^B^	0	11.12 ± 0.21 ^C^
Yeast assimilable nitrogen (mg YAN L^−1^)	553.00± 5.1 ^a^	498.75 ± 8.0 ^bA^	563.50 ± 4.4 ^a^	511.00 ± 5.2 ^bAB^	584.5 ± 4.04 ^a^	533.75 ± 6.71 ^bB^
Titratable acidity (g TA L^−1^)	1.24 ± 0.08 ^a^	2.325 ± 0.19 ^bA^	1.28 ± 0.09 ^a^	2.55 ± 0.27 ^bA^	1.54 ± 0.08 ^a^	3.11 ± 0.08 ^bB^
5-HMF (mg L^−1^)	44.57 ± 0.69 ^a^	37.57 ± 1.84 ^bA^	53.34 ±1.90 ^a^	45.72 ± 1.99 ^bB^	56.39 ± 1.47 ^a^	52.21 ± 1.58 ^bC^

TA, tartaric acid; HMF, hydroxymethylfurfural. The lowercase letters denote a significant difference for a given characteristic between the initial and final value (Student’s *t*-test, α = 0.05). The capital letters indicate a significant difference between the final values of the media assayed (Tukey’s test, α = 0.05).

**Table 4:** The initial physicochemical characteristics of the M2 media and of the corresponding final products.

Characteristic	M2–19		M2–23	
	Initial	Final	Initial	Final
Ethanol (% *v*/*v*)	0	8.040 ± 0.33 ^A^	0	8.574 ± 0.03 ^B^
Yeast assimilable nitrogen (mg YAN L^−1^)	197.4 ± 5.86 ^a^	152.25 ± 3.5 ^bA^	211.75 ± 3.50 ^a^	164.50 ± 4.04 ^bB^
Titratable acidity (g TA L^−1^)	1.09 ± 0.08 ^a^	2.36 ± 0.14 ^bA^	1.16 ± 0.08 ^a^	2.81 ± 0.14 ^bB^
5-HMF (mg L^−1^)	49.74 ± 1.07 ^a^	47.486 ± 0.57 ^bA^	59.422 ± 2.35 ^a^	57.683 ± 0.54 ^aB^

TA, tartaric acid; HMF, hydroxymethylfurfural. The lowercase letters denote a significant difference for a given characteristic between the initial and final value (Student’s *t*-test, α = 0.05). The capital letters indicate a significant difference between the final values of the media assayed (Tukey’s test, α = 0.05).

**Table 5:** Kinetic parameters for biomass growth, ethanol production, and consumption of reducing sugars in the M2–23 medium.

Logistic Model					
Biomass Growth		Ethanol Production		Consumption of Reducing Sugars	
*X_M_* (g L^−1^)	2.30 ± 0.04	*P*_M_ (g L^−1^)	62.57 ± 1.89	*S_M_* (g L^−1^)	169.3 ± 2.32
*X*_0_ (g L^−1^)	0.033 ± 0.01	*P*_0_ (g L^−1^)	9.87 ± 1.17	*S*_0_ (g L^−1^)	10.31 ± 1.52
*k* (days)	2.67 ± 0.224	*k* (days)	0.254 ± 0.02	*k* (days)	0.552 ± 0.03
R^2^	0.988	R^2^	0.9462	R^2^	0.985
SS	0.229	SS	880.6	SS	2592
Sy.x	0.116	Sy.x	4.879	Sy.x	8.37
RMSE	0.109	RMSE	4.752	RMSE	8.153
**Gompertz Model**					
**Biomass Growth**		**Ethanol Production**		**Consumption of Reducing Sugars**	
*X_M_* (g L^−1^)	2.31 ± 0.04	*P*_M_ (g L^−1^)	64.83 ± 2.02	*S_M_* (g L^−1^)	172.40 ± 2.19
*X*_0_ (g L^−1^)	0.00 ± 0.00	*P*_0_ (g L^−1^)	8.06 ± 1.12	*S*_0_ (g L^−1^)	4.17 ± 1.12
*k* (days)	1.76 ± 0.15	*k* (days)	0.17 ± 0.01	*k* (days)	0.35 ± 0.02
R^2^	0.988	R^2^	0.959	R^2^	0.989
SS	0.213	SS	663.3	SS	1940
Sy.x	0.112	Sy.x	4.234	Sy.x	7.241
RMSE	0.106	RMSE	4.124	RMSE	7.053

R^2^, square of the Pearson correlation coefficient; SS, sum of squares; Sy.x, standard deviation of the residuals; RMSE, root mean square error.

## Data Availability

The original contributions presented in the study are included in the article/Supplementary Materials, further inquiries can be directed to the corresponding author.

## Supplementary Materials

The following supporting information can be downloaded at: https://www.mdpi.com/article/10.3390/foods13121948/s1, Figure S1: Preference of the panel of judges for the mead generated by *Pichia kudriavzevii* 4A in the M2–23 versus M2–19 medium. Figure S2: Yields of *Y_X_*_/*S*_ and *Y_P_*_/*S*_ in the M2 medium with 23 °Bx of honey and *Pichia kudriavzevii* 4A as the starter culture.
