# Supplementary material for: Kinetic Evaluation of the Production of Mead from a Non-Saccharomyces Strain
Source: Foods. 2024 Jun 20;13(12):1948. doi: 10.3390/foods13121948 (PMC11203307; doi:10.3390/foods13121948)
Supplement: Supplementary file 1 [file foods-13-01948-s001.zip › foods-2992550-supplementary.pdf]

foods

MDPI

Supplementary materials

Kinetic Evaluation of the Production of Mead from a Non-*Saccharomyces* Strain

Jorge Alberto Jose-Salazar [1], Christian Bryan Ballinas-Cesatti [1], Diana Maylet Hernández-Martínez [2], Eliseo Cristiani-Urbina [1], Guiomar Melgar-Lalanne [3], and Liliana Morales-Barrera [1,*]

[1] Departamento de Ingeniería Bioquímica, Escuela Nacional de Ciencias Biológicas, Instituto Politécnico Nacional, Av. Wilfrido Massieu s/n, Unidad Profesional Adolfo López Mateos, Ciudad de México, 07738, Mexico.; jorgeajosesalazar@gmail.com (J.A.J.-S.); christian.bryan.ballinas.cesatti@hotmail.com (C.B.B.-C.); ecristianiu@yahoo.com.mx (E.C.-U.)

[2] Departamento de Biofísica, Escuela Nacional de Ciencias Biológicas, Instituto Politécnico Nacional, Prolongación de Carpio y Plan de Ayala s/n, Col. Santo Tomás, Ciudad de México, 11340, Mexico.; dhernandezmar@ipn.mx

[3] Centro de Investigaciones Biomédicas, Universidad Veracruzana, Av. Castelazo Anaya s/n, Industrial Ánimas, Xalapa 91190, Veracruz, Mexico.; gmelgar@uv.mx

* Correspondence: lmoralesb@ipn.mx; Tel.: +52-55-5729-6000 (ext. 57827)

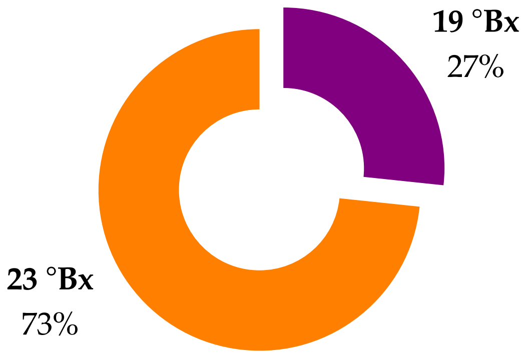

Figure S1. Preference of the panel of judges for the mead generated by *Pichia kudriavzevii* 4A in the M2-23 versus M2-19 medium.

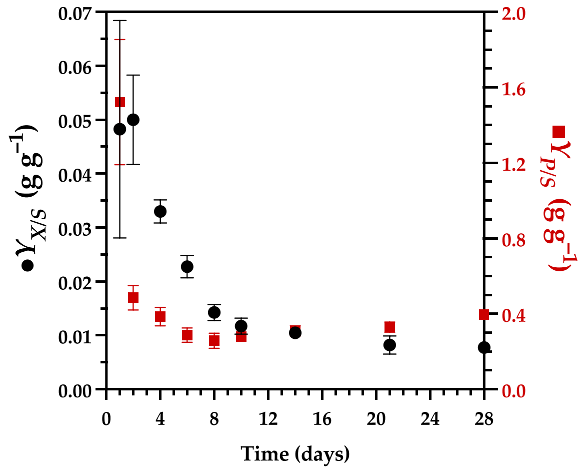

Figure S2. Yields of $Y_{X/S}$ and $Y_{P/S}$ in the M2 medium with 23 °Bx of honey and *Pichia kudriavzevii* 4A as the starter culture.
